# Comprehensive identification of *NRG1* fusions in 25,203 patients with solid tumors

**DOI:** 10.1038/s41698-025-01044-y

**Published:** 2025-07-29

**Authors:** Shui Xiang, Yiwen Zheng, Mengxiao Wang, Xuewen Liu, Xing Zhang, Dongsheng Chen, Guangxian Meng, Hongtao Xu, Xiaoxuan Wang

**Affiliations:** 1 Department of Cardiothoracic Surgery, Liuzhou Worker’s Hospital, The Fourth Affiliated Hospital of Guangxi Medical University, Liuzhou, Guangxi China; 2 Department of Pathology, The First Hospital of China Medical University, Shenyang, Liaoning China; 3 The State Key Laboratory of Neurology and Oncology Drug Development, Jiangsu Simcere Diagnostics Co., Ltd., Nanjing Simcere Medical Laboratory Science Co. Ltd., Nanjing, Jiangsu China; 4 Department of Thoracic Surgery, The Second Hospital of Dalian Medical University, Dalian, Liaoning China

**Keywords:** Cancer therapy, Tumour biomarkers

## Abstract

*NRG1* fusion is an emerging oncogenic driver, and the FDA has approved drugs for the treatment of non-small cell lung cancer and pancreatic cancer associated with *NRG1* fusions. This study retrospectively analyzed data from 25,203 patients with solid tumors who underwent next-generation sequencing (NGS) and identified 49 patients with *NRG1* fusions. The mutation profiles and actionable therapeutic targets were analyzed among patients with fusions. In this study, 0.2% (49/25,203) of patients harbored *NRG1* fusions. The frequencies of *NRG1* fusions across various cancer types were as follows: prostate cancer, 0.65%; breast cancer, 0.47%; lung cancer, 0.29%; esophageal cancer, 0.25%; colorectal cancer, 0.17%; gastric cancer, 0.13%; pancreatic cancer, 0.11%; and hepatocellular carcinoma, 0.05%). A total of 36 fusion partners were detected, among which *CD74* was predominant, accounting for 29.3% of cases. Patients with *NRG1* fusions presented a greater frequency of *FGFR1* mutations and *RET* fusions, compared with non-NRG1 fusion patients. Most lung cancer and colorectal cancer patients with *NRG1* fusions harbored FDA-approved or potential drug targets, whereas those diagnosed with breast cancer harbored fewer such targets. *NRG1* fusion-related drugs can provide additional treatment options. Our study expands the *NRG1* fusion gene landscape and provides a valuable reference for the comprehensive treatment of patients with *NRG1* fusions.

## Introduction

Neuregulin 1 (*NRG1*) is a member of the epidermal growth factor (*EGF*) family and functions as a signaling protein that regulates intercellular interactions. It is predominantly expressed in various tissues, including the brain, heart, and mammary glands, and plays a crucial role in tissue development and maturation^1,2^. As a ligand of the EGF family, *NRG1* binds to ERBB family receptor tyrosine kinases, including *ERBB2*, *ERBB3*, and *ERBB4*, to activate downstream signaling pathways involved in biological processes^3,4^. When *NRG1* undergoes fusion to form a novel fusion gene, it expresses a fusion protein on the cell membrane that retains the active EGF-like domain of *NRG1*, preserving its physiological activity. Consequently, *NRG1* fusions lead to the persistent activation of related signaling pathways, resulting in uncontrolled cell proliferation and tumorigenesis^5–8^.

Historically, numerous pharmaceutical agents have been investigated as potential treatments for patients with *NRG1* fusions. Notably, the drug zenocutuzumab, which targets *NRG1* fusions, recently received accelerated approval from the FDA, marking a significant advance as the first targeted therapy for patients with *NRG1* fusions.

*NRG1* fusions represent important molecular events that are implicated in the tumorigenesis of various cancers. However, their occurrence is rare, with a frequency of less than 1% across tumors. The highest frequency of *NRG1* fusions is observed in lung cancer. Nevertheless, they are also detected in other cancers. *NRG1* fusions involve multiple fusion partner genes, with *CD74*, *ATP1B1*, and *SDC4* being the most common^5^. In lung cancer specifically, several *NRG1* fusion partners, including *CD74*, *ATP1B1*, *SLC3A2*, *SDC4*, *RBPMS*, and *WRN*, have been identified^9–11^. Other *NRG1* fusion partners, including *POMK*, *APP*, *CDH6*, *ATP1B1*, and *CLU*, have also been found in various solid tumors^12–14^.

This study aimed to comprehensively investigate the frequency, structural characteristics, co-occurring mutations, and prognostic impact of *NRG1* fusions. Using next-generation sequencing (NGS), we conducted molecular profiling of 25,203 solid tumor samples from Chinese patients and analyzed data from 49 patients with *NRG1* fusions, obtaining valuable clinical insights and identifying therapeutic opportunities for patients harboring *NRG1* fusions in solid tumors.

## Results

### The frequency of *NRG1* fusions across solid tumor types

We collected next-generation sequencing (NGS) data from tumor samples from 25,203 patients across 15 types of solid tumors (Table 1). Among these patients, 49 patients with *NRG1* fusions were identified, resulting in an overall frequency of 0.2%. Lung cancer (49.4%) was the most prevalent tumor type among all patients included in this study, followed by colorectal cancer (11.1%), liver cancer (8.5%), gastric cancer (6.0%), pancreatic cancer (3.7%), sarcoma (2.6%), breast cancer (2.5%), glioma (2.1%), head and neck cancer (1.7%), cervical cancer (1.6%), esophageal cancer (1.6%), cholangiocarcinoma (1.1%), prostate cancer (0.6%), medulloblastoma (0.1%) and other tumors. In the total cohort, 57.7% of patients were male, and 21% were classified as stage IV at diagnosis.

**Table 1:** Baseline characteristics of patients with *NRG1* fusion and non-fusion

Characteristic	Fusion (n = 49)	Non-fusion (n = 25,154)
Sex, n (%)		
Male	18 (36.7%)	14522 (57.7%)
Female	31 (63.3%)	10632 (42.3%)
Age, n (%)		
>60	33 (67.3%)	12865 (51.1%)
≤60	16 (32.7%)	12289 (48.9%)
Stage, n (%)		
I	0	480 (1.9%)
II	2 (4.1%)	843 (3.4%)
III	0	1646 (6.5%)
IV	8 (16.3%)	5275 (21.0%)
Unknown	39 (79.6%)	16910 (67.2%)
Diagnosis, n (%)		
Lung cancer	36 (73.5%)	12422 (49.4%)
Colorectal cancer	3 (6.1%)	2799 (11.1%)
Breast cancer	3 (6.1%)	632 (2.5%)
Gastric cancer	2 (4.1%)	1510 (6.0%)
Liver cancer	1 (2.0%)	2137 (8.5%)
Esophageal cancer	1 (2.0%)	393 (1.6%)
Pancreatic cancer	1 (2.0%)	932 (3.7%)
Prostate cancer	1 (2.0%)	154 (0.6%)
Medulloblastoma	1 (2.0%)	12 (0.1%)
Sarcomas	0	662 (2.6%)
Glioma	0	516 (2.1%)
Head and neck cancer	0	432 (1.7%)
Cervical cancer	0	398 (1.6%)
Cholangiocarcinoma	0	272 (1.1%)
Other	0	1883 (7.5%)

**Fig. 1:**
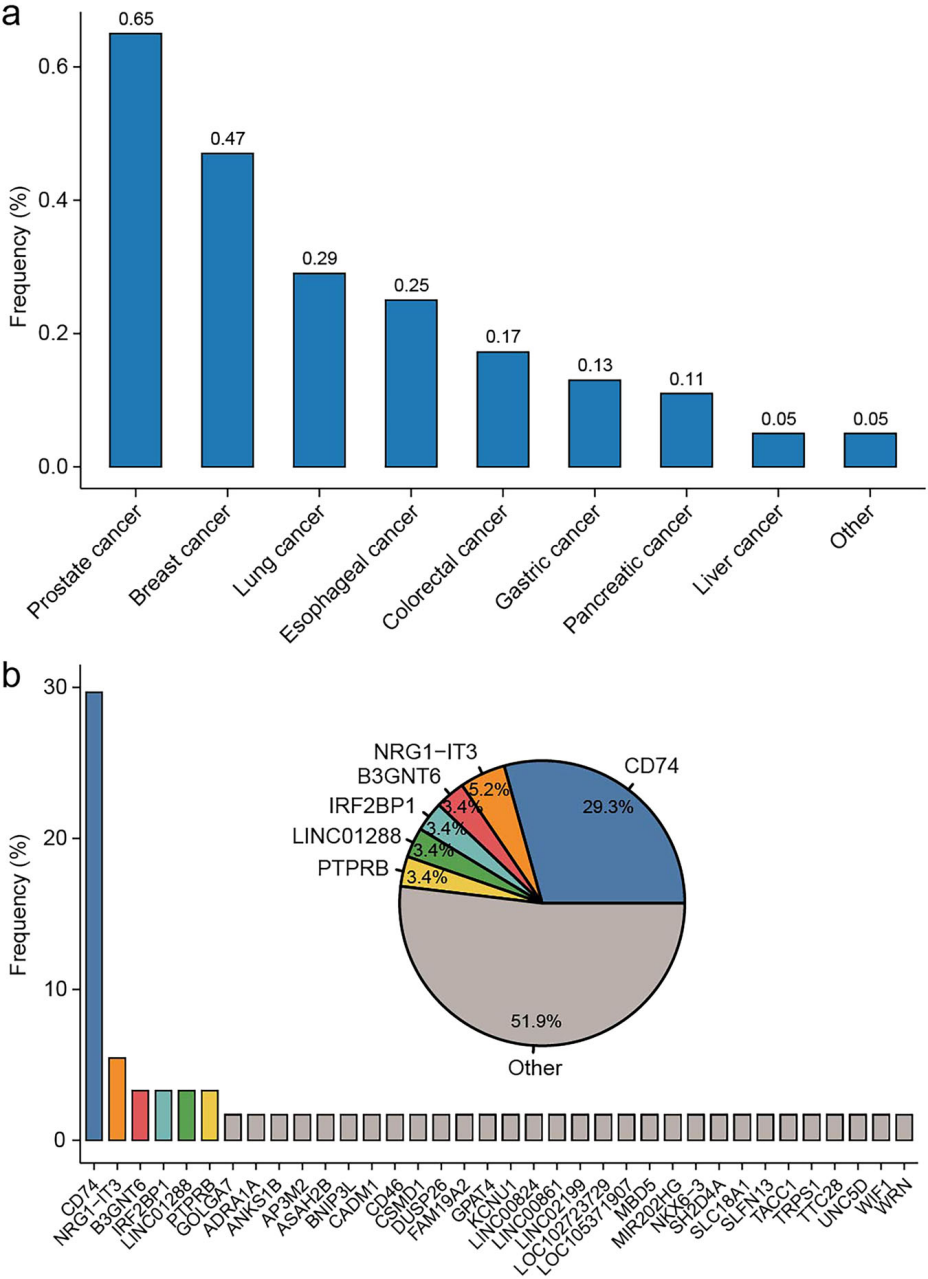
Frequencies of *NRG1* fusion and fusion partners. **a** Frequencies of *NRG1* fusion in different tumor types. **b** Frequencies of fusion partners identified in the study.

A total of 36 *NRG1* fusion events were identified in 12,458 lung cancer patients, reflecting a frequency of 0.29% within this group (Fig. 1a). Notably, the frequency of *NRG1* fusion was greater in several other tumor types than in lung cancer, with values of 0.65% in prostate cancer and 0.47% in breast cancer. In addition, the observed frequencies of *NRG1* fusions in other tumor types were 0.25% in esophageal cancer, 0.17% in colorectal cancer, 0.13% in gastric cancer, 0.11% in pancreatic cancer, and 0.05% in liver cancer (Fig. 1a). We did not capture *NRG1* fusion events in sarcoma and glioma. Compared with male patients, female patients were more likely to present with *NRG1* fusions (*p* = 0.003); however, there were more male patients overall in the cohort.

### The genomic landscape of *NRG1* fusions

**Fig. 2:**
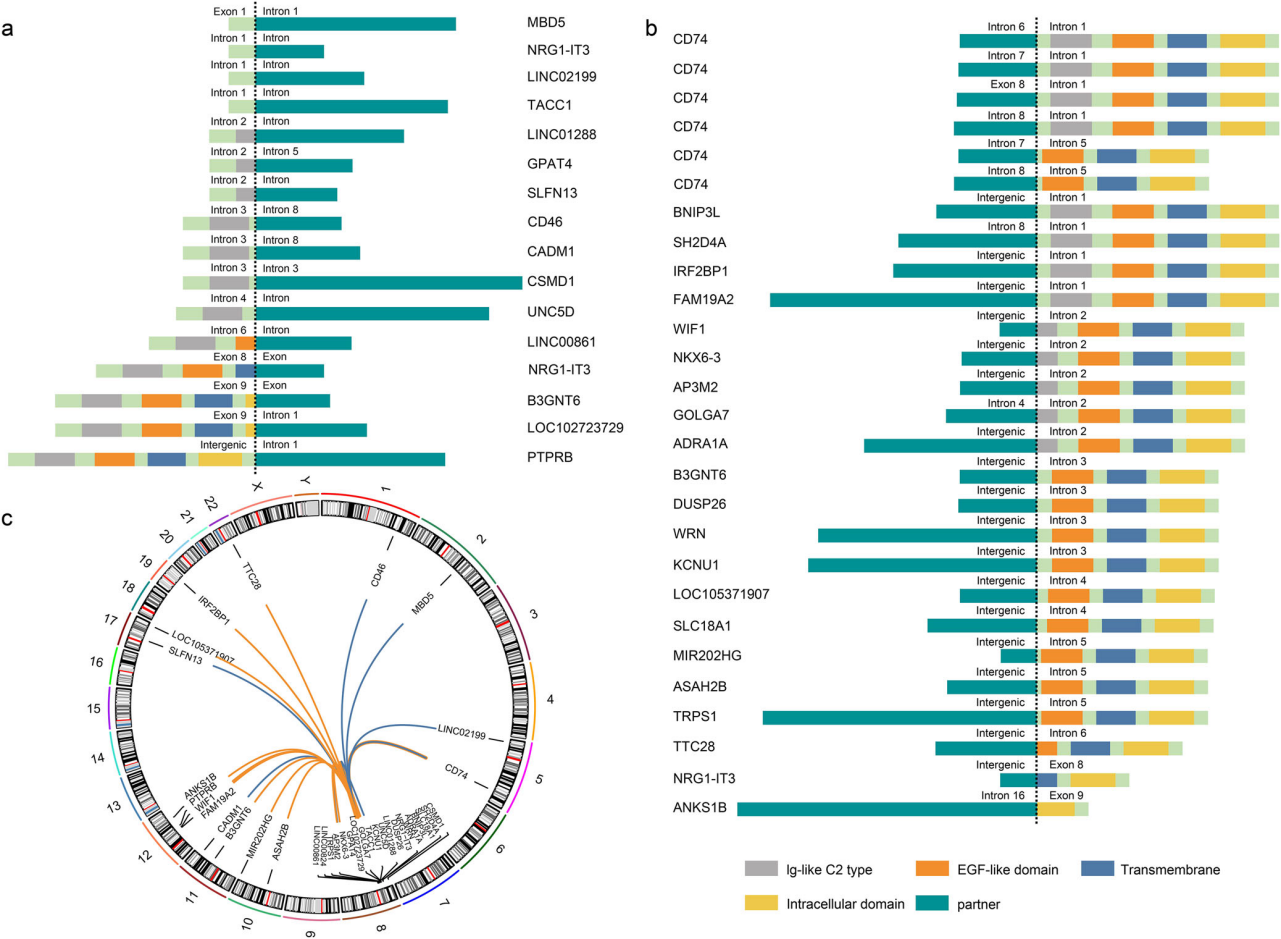
Schematic diagram of *NRG1* fusions. **a** *NRG1* type I fusion, 5′ *NRG1* joined to 3′ partners. **b** *NRG1* type II fusion, with 5′ partners joined to 3′ *NRG1*. **c** locations of the fusion partners on the chromosome. The blue and orange lines indicate *NRG1* type I fusions and type II fusions, respectively.

*NRG1* consists of a total of 13 exons, with a functional EGF-like domain in exons 6 and 7. Exon 8 encodes a transmembrane domain. Gene fusions can occur at both the 5′ end and the 3′ end of *NRG1*. In the present study, we identified a total of 36 fusion partners associated with *NRG1* (Fig. 1b). The most frequent partner was *CD74* (29.3%), followed by *NRG1-IT3* (5.2%), *B3GNT6* (3.4%), *IRG2BP1* (3.4%), *LINC01288* (3.4%), and *PTPRB* (3.4%) (Fig. 1b). Among the detected partners, 16 fused to the 3′ end of *NRG1*, and 11 of these fusions disrupted the EGF-like domain (Fig. 2a). In contrast, 22 partners fused to the 5′ end of *NRG1*, and only three of these fusions compromised the integrity of the EGF-like domain (Fig. 2b). Most of the identified fusion partners were located on the same chromosome as *NRG1*, namely, chromosome 8. Other fusion partner locations include chromosomes 1, 2, 5, 10, 11, 12, 17, 19, and 22 (Fig. 2c).

### Co-occurrence of gene mutations and *NRG1* fusions

**Fig. 3:**
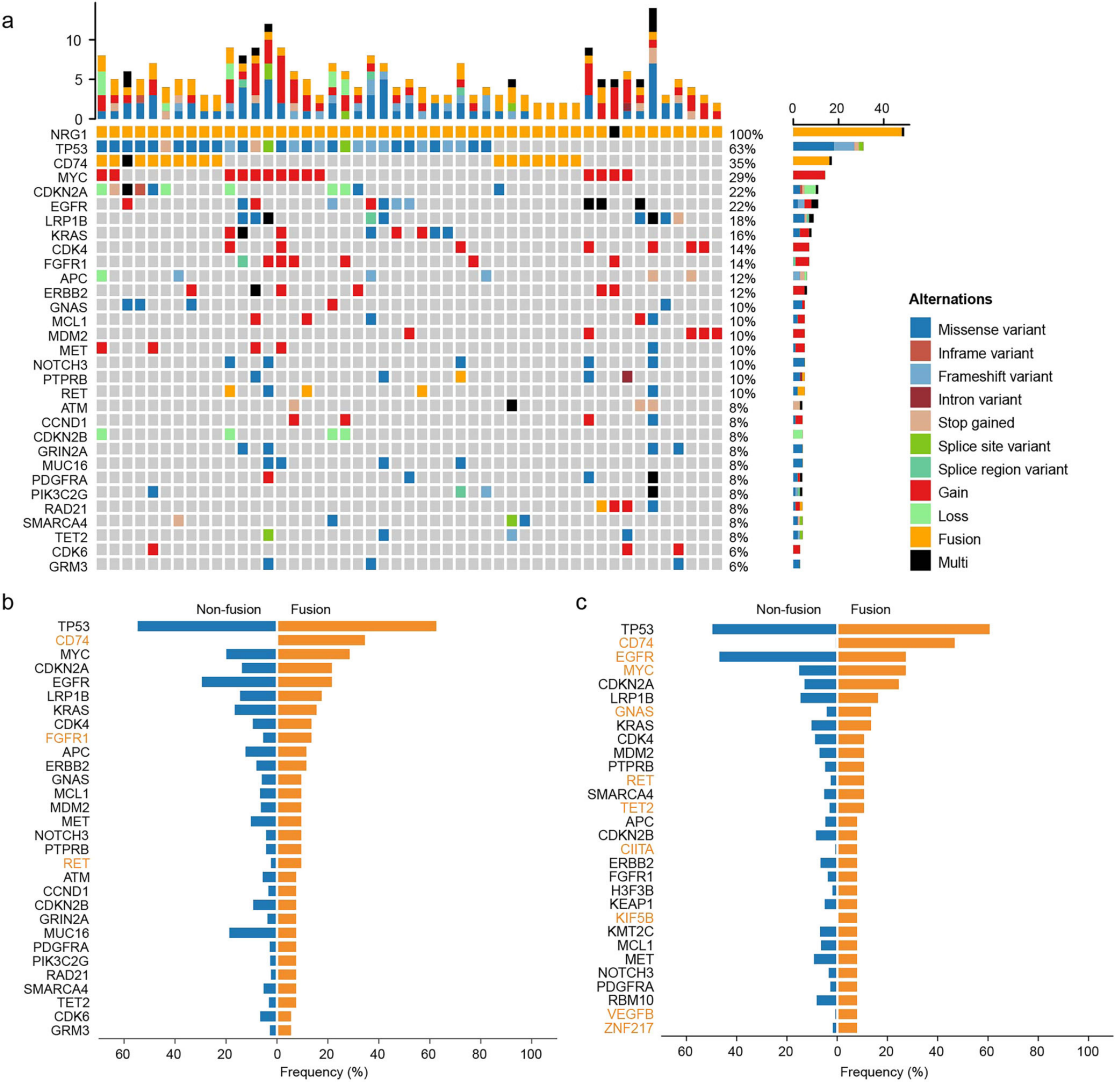
Mutational landscape of patients with *NRG1* fusions. **a** Heatmap of the comutations of 49 patients with *NRG1* fusions. **b** Comparison of mutation frequency between patients with *NRG1*-fusion and *NRG1*-nonfusion solid tumors. **c** Comparison of mutation frequency between *NRG1* fusion and *NRG1*-nonfusion lung cancer patients. The genes highlighted in orange signify a significant mutation frequency between fusion and non-fusion. Chi-square test or Fisher’s exact test, P < 0.05.

The genomic alterations that co-occurred with *NRG1* fusion were analyzed across all the samples. The most frequently mutated genes included *TP53* (63%), *CD74* (35%), *MYC* (29%), *CDKN2A* (22%), and *EGFR* (22%) (Fig. 3a). Patients with *NRG1* fusions presented significantly higher mutation frequencies of *CD74* (*p* < 0.001), *FGFR1* (*p* = 0.028), and *RET* (*p* = 0.007) than did patients without *NRG1* fusions (Fig. 3b). In the context of lung cancer, significantly more gene mutation events, including mutations in *CD74* (*p* < 0.001), *EGFR* (*p* = 0.019), *MYC* (*p* = 0.045), *GNAS* (*p* = 0.025), *RET* (*p* = 0.019)*, TET2* (*p* = 0.044), *CIITA* (*p* = 0.009), *KIF5B* (*p* = 0.003), *VEGFB* (*p* = 0.01), and *ZNF217* (*p* = 0.017), occurred in patients with *NRG1* fusions (Fig. 3c).

### Tumor biomarkers in patients with *NRG1* fusions

**Fig. 4:**
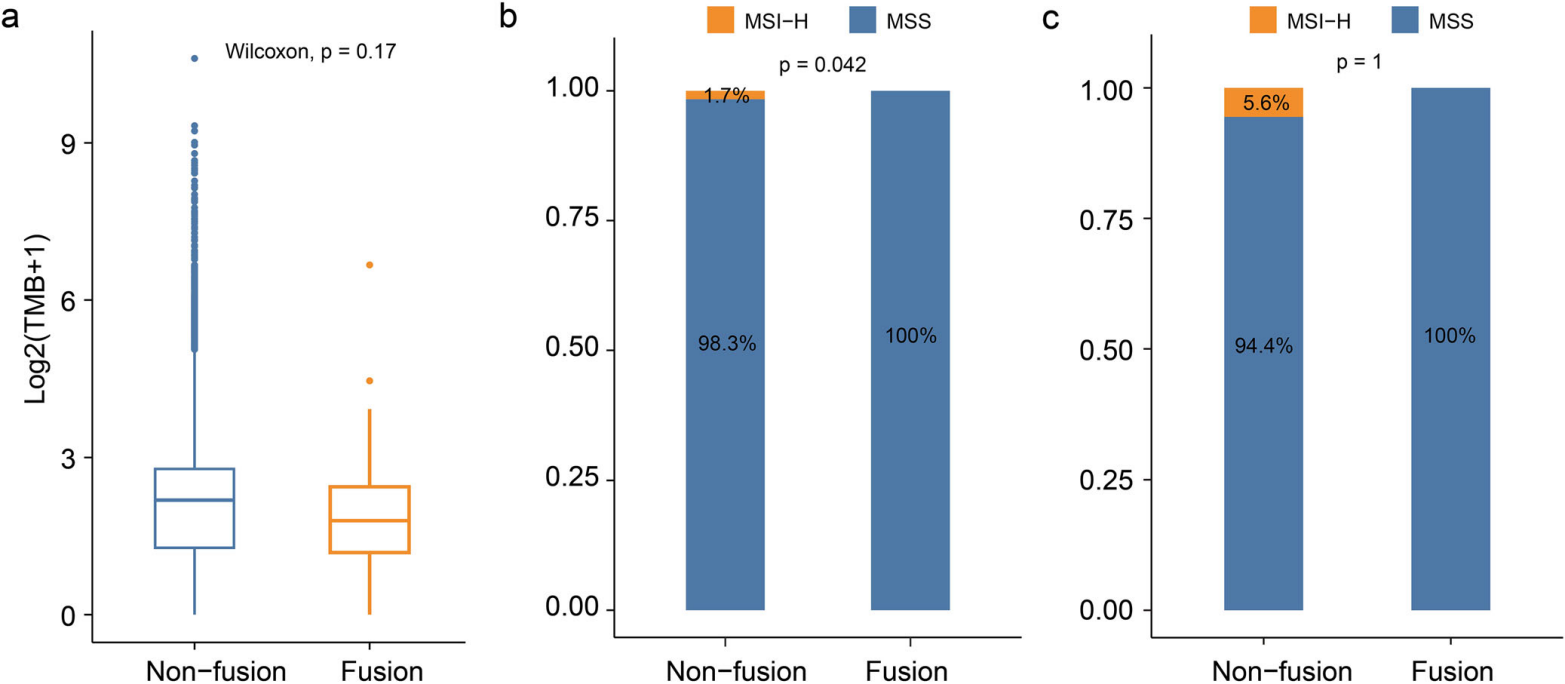
Immunotherapy-related biomarkers in patients with *NRG1* fusion. **a** comparison of TMB between fusion and non-fusion. The centre of the box is represented by the median value. Wilcoxon test, *P* < 0.05. Comparison of MSI status between fusion and non-fusion in pan-cancer (**b**) and gastrointestinal tumors (**c**). Chi-square test or Fisher’s exact test, *P* < 0.05.

We further compared the differences in pancancer biomarkers between patients with and without *NRG1* fusion. Our analysis revealed no significant difference in TMB between the two groups (*p* = 0.17, Fig. 4a). The variation in TMB across both groups suggests that additional factors beyond *NRG1* fusion status may influence mutational profiles. Notably, according to some studies, more than 80% of microsatellite instability-high (MSI-H) tumors are classified as TMB-high (TMB-H); however, only 18.3% of TMB-H tumors are MSI-H^15^. Nevertheless, we observed a significant difference in the frequency of MSI-H tumors between the two groups. Patients with NRG1 fusion presented a markedly lower proportion of MSI-H than did those without this fusion (*p* = 0.042, Fig. 4b). Specifically, all patients with *NRG1* fusions were found to be microsatellite stable (MSS). These findings suggest that patients with NRG1 fusions may have a poor response to immune monotherapy. Gastrointestinal tumors, for which MSI status is frequently utilized clinically as a guide for treatment, were analyzed independently. However, due to the limited number of *NRG1*-fusion-positive cases in this subset, no statistically significant associations with MSI status could be established (*p* = 1, Fig. 4c).

### *NRG1* fusions as potential therapeutic targets

The *NRG1* fusion drug zenocutuzumab has been approved by the FDA, offering a new treatment option for patients. To explore additional potential therapeutic targets and facilitate comprehensive treatment decisions, we analyzed actionable mutation profiles in patients with *NRG1* fusions across lung cancer, colorectal cancer, and breast cancer.

**Fig. 5:**
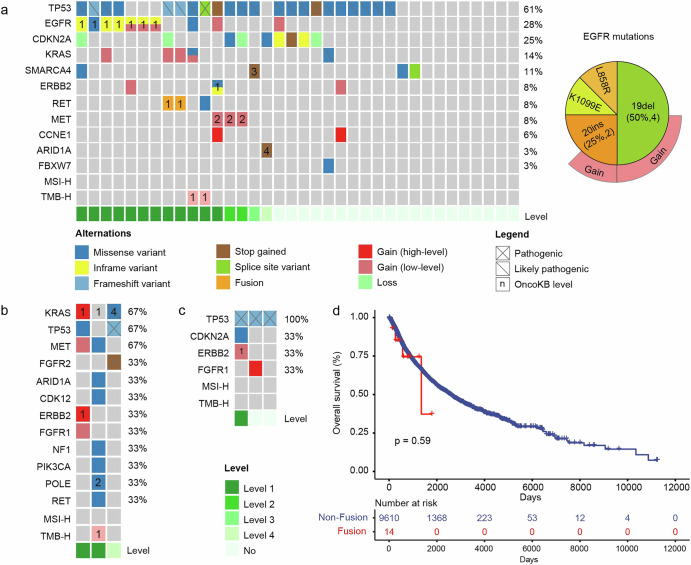
Therapeutic or potential targets in patients with *NRG1* fusion. Exploration of therapeutic or potential targets in *NRG1* fusion patients with lung cancer (**a**), colorectal cancer (**b**), and breast cancer (**c**). The numerical values displayed on the heatmap represent the level of the gene mutation annotated in the OncoKB database. **d** Kaplan‒Meier curve showing overall survival between *NRG1*-fusion patients and nonfusion patients in the TCGA cohort.

In the lung cancer cohort, nearly half of the patients (15/36) with *NRG1* fusion lung cancer had targets for approved/potential drugs that could be treated with other targeted agents (Fig. 5a). Specifically, seven patients with *EGFR* mutations had the potential to benefit from more than a dozen approved EGFR-targeting drugs, such as gefitinib and osimertinib. The *EGFR* mutations identified include exon 19 deletions (19del, n = 4), exon 20 insertions (20ins, n = 2), and L858R (n = 1). One patient with *ERBB2* amplification could be treated with ERBB2-targeted therapies. Additionally, approved targeted agents for *RET* fusions were available for two patients with *RET* fusions, and patients with *MET* amplification were potentially suitable for Met-targeting drugs such as sevotinib and britinib. The presence of pathogenic mutations in *SMARCA4* and *ARID1A* opens further avenues for targeted therapies, particularly given the growing interest in drugs that inhibit these pathways.

The treatment strategy for patients with colorectal cancer can be determined by *RAS/RAF* mutation status (Fig. 5b). Notably, we identified actionable *ERBB2* mutations (n = 1) indicating potential benefit from targeted inhibitors. Furthermore, one patient exhibited TMB-H status, suggesting possible eligibility for immune checkpoint inhibitor therapy. Within the cohort, three patients diagnosed with breast cancer were found to have *NRG1* fusions. However, the number of approved or potential drug targets in breast cancer patients is limited, and only 1 patient with *ERBB2* amplification had a relevant target (Fig. 5c). These results indicate the potential to further expand the population benefiting from *ERBB2* inhibitors.

### Prognosis of patients with *NRG1* fusion

Preliminary studies have indicated a disappointing prognosis for patients diagnosed with *NRG1* fusion tumors^16,17^. We conducted a survival analysis comparing patients with and without *NRG1* fusion, utilizing data extracted from the TCGA database. The findings revealed no significant difference in overall survival across the pan-cancer cohort (Fig. 5d). However, owing to the limited number of patients with *NRG1* fusions across each cancer type, our study did not investigate the impact of *NRG1* fusions on prognosis within specific cancer types.

## Discussion

To demonstrate the landscape of *NRG1* fusion, we retrospectively analyzed data from more than 25,000 samples. In this study, *NRG1* fusions were observed in lung cancer, colorectal cancer, breast cancer, gastric cancer, liver cancer, esophageal cancer, pancreatic cancer, prostate cancer, and medulloblastoma. The frequencies of *NRG1* fusion in prostate cancer, breast cancer, lung cancer, and esophageal cancer were greater than the overall frequency of 0.2%. In addition, the frequencies of *NRG1* fusion in colorectal cancer, gastric cancer, pancreatic cancer, and liver cancer were lower than the overall frequency. Unexpectedly, no *NRG1* fusion events were identified in sarcoma or glioma, although the sample numbers of both tumor types were greater than 0.2%. Interestingly, multiple types of gene fusions have been reported to be abundant in sarcoma^18–21^. The scarcity of *NRG1* fusions in sarcoma indicates that the development of NRG1 fusion-targeted therapy for sarcoma may not be high-priority.

The positions of most breakpoints of *NRG1* fusions ranged from intron 1 to intron 5. Whether the EGF-like domain is disrupted depends on which end of *NRG1* is retained after fusion. The typical fusions usually contain the 3′ end of *NRG1*^11,12^. In this study, we detected several cases that contained the 5′ end of *NRG1*. Regardless of which end of *NRG1* the partners fused into, the functions of the fusions remain to be characterized. In this study, *CD74* was identified as the most frequent fusion partner of *NRG1*, which was consistent with previous findings. *CD74* encodes a multifunctional protein involved in diverse biological processes^22–24^. As a surface receptor, *CD74* can activate pathways involved in the proliferation and survival of tumors^23,25^. As a high-affinity surface receptor of MIF, *CD74* participates in the regulation of antigen presentation in the immune response^26–28^.

To elucidate the potential functions of these fusion events in oncology, we analyzed the co-occurrence of gene mutations in patients with *NRG1* fusions. *TP53* mutation occurred in most *NRG1* fusion cases. However, the difference in the frequency of *TP53* mutation between patients with and without *NRG1* fusion was not significant. Regardless of the tumor type, the mutation frequencies of *CD74*, *FGFR1*, and *RET* were significantly greater in patients with *NRG1* fusion than in patients without *NRG1* fusion. The co-occurrence of *NRG1* fusion and oncogenes, such as *FGFR1* and *RET,* might provide a new option for physicians in making treatment decisions. Alterations in more genes, including *CD74*, *EGFR*, *MYC*, *GNAS*, *RET*, *TET2*, *CIITA*, *KIF5B*, *VEGFB*, and *ZNF217*, were found to cooccur with *NRG1* fusions in lung cancer patients.

Prior studies have documented that low TMB is associated with reduced efficacy of immunotherapy^29,30^. The efficacy of immunotherapy relies largely on the ability of immune cells to recognize cancer cell-specific antigens. Consequently, cancer cells with a greater number of genetic mutations are theoretically more likely to be recognized by immune cells because of producing a greater number of neoantigens^30–32^. A large multicenter clinical trial, eNRGy1, demonstrated that patients with lung cancer who exhibit an *NRG1* fusion with low PD-L1 expression (28%) and low TMB demonstrate a reduced response to immunotherapy^17^. The findings from our analysis reveal important insights into the tumor biology associated with *NRG1* fusions. The lack of a significant increase in TMB in the *NRG1* fusion cohort suggests that these tumors may not follow the typical mutational patterns observed in other genomic alterations that result in a greater mutational burden. MSI-H and *NRG1* fusion are two distinct markers in tumors that may coexist in some cases and may affect patient response to therapy when coexisting^33^. The presence of MSI-H in non-*NRG1* fusion patients suggests the potential for distinct oncogenic mechanisms in *NRG1* fusion patients versus nonfusion patients, which may influence treatment response. These findings emphasize the need for further research on more effective targeted therapies for *NRG1* fusion patients.

Patients with *NRG1* fusions usually respond poorly to standard chemotherapy, immunochemotherapy, or ICIs (such as PD-1/L1 monoclonal antibodies)^34,35^. A range of pharmaceutical agents are currently available, with a primary focus on targeted therapy drugs and monoclonal antibodies. One of these medications is a small-molecule tyrosine kinase inhibitor (TKI) that targets the EGFR/HER2 pathway, while the other is a large-molecule monoclonal antibody that binds to HER2/HER3^36–38^. Recently, zenocutuzumab was approved for the treatment of adult patients with advanced unresectable or metastatic non-small cell lung or pancreatic cancer who are positive for *NRG1* fusion and who have experienced disease progression despite other treatments. This is the first targeted drug approved by the FDA for the treatment of patients with *NRG1* fusion-carrying non-small cell lung cancer or pancreatic cancer^38,39^.

Our results revealed multiple potential drug targets in *NRG1* fusion patients with lung cancer, colorectal cancer, and breast cancer. According to the findings of previous studies, the occurrence of *NRG1* fusion in solid tumor patients has been demonstrated to be associated with a favorable response to the clinical implementation of Erb family inhibitor treatment, which has been shown to result in improved prognoses and survival outcomes^36,40,41^. The emergence of *RTK* fusions is one of the mechanisms of EGFR-TKI drug resistance. Dual inhibition of EGFR-RTK is safe and effective in patients with targetable *RTK* fusions after the progression of EGFR-TKIs^42^. In our study, patients with colorectal cancer or breast cancer showed the potential for response to drugs targeting *ERBB2*. Drugs targeting the ERBB2/3 kinase domain can inhibit the continued activation of the ERBB2/3 pathway via *NRG1* gene fusion, thereby inhibiting cancer progression. In addition to the previously mentioned TKI drugs, a variety of monoclonal antibodies, including trastuzumab and pertuzumab, have been shown to inhibit dimerization and promote antibody-dependent cellular toxicity by binding to the ERBB2 extracellular domain. Other therapeutic agents in this class include seribantumab, lumretuzumab, elgemtumab, GSK2849330, KTN3379, and AV-203, which target *ERBB3*. Moreover, ADC drugs, such as patritumab and zenocutuzumab, have demonstrated therapeutic potential in clinical trials^35,43,44^. Concurrently, the presence of *KRAS* mutations in colorectal cancer patients suggests the potential benefit of targeted drugs. This highlights the need for personalized treatment strategies based on individual genetic profiles. Tumor genetic testing facilitates the identification of novel drug targets for patients with *NRG1* fusions.

However, the limitation of this study is that only DNA sequencing was used to characterize the map, and the lack of multiomics joint verification may lead to incomplete *NRG1* fusion detection. A combination of DNA and RNA testing may be necessary to obtain more comprehensive information and further refine the research findings. Understanding the broader context of *NRG1* fusions within the landscape of oncogenic drivers may ultimately enhance personalized treatment strategies and improve patient outcomes. Additionally, the absence of detailed treatment history limits our ability to assess therapy-induced changes in the mutational landscape.

In conclusion, our findings indicate that tumors harboring *NRG1* fusions have distinct genetic and molecular characteristics. Understanding these differences is critical for developing personalized treatment strategies and improving the clinical prognosis of patients with these specific genetic alterations.

## Methods

### Patient cohort

From November 2021 to September 2022, a total of 25,203 patients with 15 cancer types who underwent NGS were enrolled in this retrospective study. Targeted sequencing using either a 539-gene or 551-gene panel to identify a comprehensive genomic profile was performed by Simcere Diagnostics Co., Ltd. (Nanjing, China), which is certified by the Clinical Laboratory Improvement Amendments (CLIA), College of American Pathologists (CAP), and ISO15189. The clinical data collected included age, sex, tumor stage, and tumor type. This study was carried out in accordance with the Declaration of Helsinki. Approval was obtained from the Ethics Committee of Liuzhou Worker’s Hospital and The First Hospital of China Medical University. The Ethics Committee granted a waiver of informed consent based on review and determination that this research meets the following requirements: (i) this research is a observational study; (ii) the personal information of the subjects is strictly confidential; (iii) the waiver will not adversely affect the rights and welfare of the subjects.

### Library preparation and NGS sequencing

DNA was extracted from patient samples using the DNeasy Tissue Kit. The KAPA Library Preparation Kit was utilized for library construction, while the Invitrogen Qubit 4.0 was used to evaluate the library concentration. Sequencing was performed with an average depth of 1500x using an Illumina NextSeq 550 or NovaSeq 6000 system.

### Variant calling and bioinformatics analysis

Fastp (v.2·20·0)^45^ was used to trim adapters and filter low-quality reads. BWA-mem (v.0·7·17)^46^ was then used to align the cleaned paired-end reads to the human reference genome (hg19). Single nucleotide variants (SNVs) and insertion‒deletion variants (indels) were identified using the tools VarDict (v.1·5·7)^47^ and InterVar^48^. Copy number variants (CNVs) were detected using CNVkit (dx1·1), and FACTERA (v1·4·4) was utilized to call fusion events. High-level amplification was defined as ≥ 6 copies^49^. A total of 334 homopolymer repeat loci were selected to determine microsatellite instability (MSI) status, with the cutoff for MSI-high (MSI-H) defined as 0.15. The tumor mutational burden (TMB) was calculated by summing all the SNVs and indels in the coding region of the targeted genes, with TMB-high (TMB-H) defined as ≥ 10 mut/Mb.

### Therapeutic target analysis

OncoKB (v4.24), a precision oncology knowledge database, was used to identify therapeutic or potential targets^50,51^. Level 1 indicates an FDA-recognized biomarker for predicting drug response. Level 2 includes standard-of-care biomarkers that are predictive of the response to an FDA-approved drug. Level 3 reflects compelling clinical evidence or investigational biomarkers. Level 4 indicates compelling biological evidence.

### Statistical analysis

All the data were analyzed using R version 4.3.2. The genomic landscape was visualized using the R package ComplexHeatmap (v2.18.0). The chromosome circos plot was generated with the R package RCircos (v1.2.2). Clinical and genomic data from The Cancer Genome Atlas (TCGA) were retrieved from cBioPortal. The Kaplan‒Meier curves were analyzed with the log-rank test using the R packages Survival (v3.5.7) and Survminer (v0.4.9). Differences in TMB between groups were assessed using the Wilcoxon test, whereas categorical variables were analyzed using either the chi-square test or Fisher’s exact test. *P* < 0.05 was considered to indicate statistical significance.

## Data Availability

The datasets generated and/or analysed during the current study are not publicly available due to policies and regulations.
